# Renin–angiotensin system blockade after transcatheter aortic valve replacement (TAVR) improves intermediate survival

**DOI:** 10.15171/jcvtr.2019.30

**Published:** 2019-08-13

**Authors:** Brent Klinkhammer

**Affiliations:** University of Nebraska Medical Center, Omaha, NE 68198, USA

**Keywords:** Antihypertensive Agents, TAVR, ARB, ACE inhibitor

## Abstract

***Introduction:*** Hypertension is common in patients with severe aortic stenosis undertaking transcatheter aortic valve replacement (TAVR). Renin–angiotensin system (RAS) blockade therapy with angiotensin-converting enzyme inhibitors (ACEIs) or angiotensin II receptor blockers (ARBs) has recently been associated with improved outcomes after surgical aortic valve replacement and TAVR, but it is unknown if these findings apply to a more rural patient population.

***Methods:*** A retrospective cohort study of 169 patients with at least 1 year of post-TAVR follow-up at a single predominantly rural US center was performed to determine if RAS blockade after TAVR affects short- and long-term outcomes. Seventy-one patients were on an ACEI or ARB at the time of TAVR and at 1 year post-TAVR follow-up. Fisher’s exact test was used for categorical data and t-test/ANOVA was used to determine the statistical significance of continuous variables.

***Results:*** In a well-matched cohort, RAS blockade therapy post-TAVR was associated with significantly improved overall survival at 2 years (95% vs. 79%, P = 0.042). RAS blockade was also associated with a trend towards decreased heart failure exacerbations in the first year after TAVR, which was statistically significant in the 30 days to 6 months timeframe after TAVR (8% vs. 21%, *P* = 0.032).

***Conclusion:*** In a rural patient population, RAS blockade after TAVR is associated with improved overall survival and a trend towards decreased heart failure exacerbations. This study builds upon previous studies and suggests that TAVR should be considered a compelling indication for these agents.

## Introduction

Angiotensin-converting enzyme inhibitors (ACEIs) have been widely prescribed worldwide and have been associated with reduction in cardiovascular mortality in a large range of patients with cardiovascular disease, and in patients at risk for negative cardiovascular outcomes.^1-5^ Likewise, angiotensin II receptor blockers (ARBs) may provide similar results to ACEIs for certain indications, although the clinical evidence is not as robust.^6^ Previous studies have shown that the use of either ACEIs or ARBs following surgical aortic valve replacement are associated with improved survival out to 10 years.^7^

However, recent advances in transcatheter aortic valve replacement (TAVR) and data showing the noninferiority of TAVR in patient with in patients at low and intermediate surgical risk, has made TAVR the aortic valve replacement procedure of choice for many patients.^8,9^ Recent studies have indicated an association with RAS blockade and overall survival in patients after TAVR, however the external validity of these findings remain in question.^10^ It is well known that patients in rural areas have less access to advanced cardiovascular care and nephrology, which complicates RAS blockade therapy.^11,12^ Furthermore, the prevalence of cardiovascular risk factors and hypertension is higher in rural areas, indicating that this may be a significant area of growth for TAVR in the future.^11^ In this study, we hypothesized that RAS blockade therapy with ACEIs or ARBs would result in similar improved outcomes in patients after TAVR in a predominately rural patient population.

## Materials and Methods

A retrospective cohort study was conducted using data from a single US center in a predominantly rural area. Chart review of 342 consecutive patients, who underwent a transcatheter aortic valve replacement (TAVR) for severe aortic stenosis at Sanford Health in Fargo, ND from 8/10/2012 to 11/15/2016, was performed. Severe aortic stenosis was defined as an aortic valve area less than 1 cm^2^. The last date of data acquisition was 1/4/2017. Patients with less than 1 year of post-TAVR follow-up were excluded. To regulate and retrospectively adjust for drug exposure in the RAS blockade cohort, the entire cohort was divided in two groups in which subjects on an ACEI or ARB at the time of TAVR and remained on an ACEI or ARB at a routine outpatient visit at 1 year post-TAVR were included in one cohort, while all other patients with at least 1 year of post-TAVR follow-up were designated as controls. Primary outcome was overall survival at 2 years post-TAVR. Secondary outcomes included major adverse cardiovascular and cerebrovascular events (MACCE) defined as death from any cause, myocardial infarction, rehospitalization, or stroke, cardiovascular mortality, myocardial infarction, all-cause hospital readmission, procedural complications, stroke/TIA, all-cause mortality, or heart failure exacerbations as defined below. Outcomes were defined in accordance with the standardized endpoint definitions of the Valve Academic Research Consortium-2.^13^ Heart failure exacerbation was defined as a change in symptoms of consistent with heart failure necessitating additional pharmacotherapy or hospitalization.

Informed consent was not required for inclusion in this study due to its nature and the absence of any direct interventions. This study protocol received dual IRB approval from the University of North Dakota IRB and from the Sanford Health IRB. SPSS 23.0 for Windows was used to analyze demographic and clinical characteristics of patients. Frequencies and relative percentages were computed for each categorical variable. Fisher’s exact test was performed to determine statistical significance of categorical data and t-test/ANOVA was used to determine the statistical significance of continuous variables. All P-values were 2-sided, and *P* values < 0.05 were considered significant.

## Results

Of the 169 patients that met study criteria for inclusion, 71 were prescribed RAS blockade therapy at the time of TAVR and remained on an ACEI or ARB at the time of routine 1 year post-TAVR follow-up. Baseline characteristics for both groups are given in Table 1. Overall, the cohorts were well matched, however statistically significant differences were noted preprocedural hypertension and diabetes mellitus. There were no significant differences in non-RAS blockade cardiovascular pharmacology. There was a high prevalence of comorbidities in both groups, including an 84% prevalence of hypertension in the entire cohort. Mean age of the entire cohort was 79 years of age. Procedural characteristics for both groups are given in Table 2. There were no statistical differences in the specific type of valve implanted or TAVR approach. Transfemoral TAVR was extensively utilized in both cohorts. Pre and postprocedural echocardiographic data is given in Table 3. A significant increase in severe mitral regurgitation at baseline was noted in the control group which was not sustained after the procedure. No significant differences in LV ejection fraction or mean aortic gradient was noted. Finally, the primary and secondary outcomes data for this study is given in Table 4, which demonstrates improved overall survival in the RAS blockade cohort in comparison to the cohort group at 2 years (95% vs. 79%, *P* =0.042). Overall survival for the entire study cohort was 85.1% at 2 years. The results of this study also demonstrate a trend towards decreased heart failure exacerbations in the first year after TAVR in the ACEI/ARB cohort, which was statistically significant in the 30 days to 6 months timeframe (8% vs. 21%, *P* =0.032).

**Table 1:** Baseline Characteristics

	**RAAS (n=71)**	**Control (n=98)**	**P-value**
Age	77.8 (7.9)	80.1 (7.5)	0.057
Male sex	56 (40)	61 (60)	0.531
BMI	31.0 (6.7)	29.9 (6.3)	0.302
Caucasian race	97 (69)	99 (97)	0.573
EuroSCORE (%)	8.63 (6.32)	10.43 (7.18)	0.101
Preprocedural HTN	96 (68)	77 (75)	<0.001
Preprocedural CAD	75 (53)	77 (75)	0.856
Baseline ejection fraction <40%	21 (15)	11 (11)	0.088
Preprocedural NYHA Class III OR IV symptoms	42 (30)	50 (49)	0.351
Preprocedural DM	51 (36)	32 (31)	0.017
Prior Stroke/TIA	10 (7)	13 (13)	0.631
Preprocedural atrial fibrillation	27 (19)	30 (29)	0.732
Preprocedural serum creatinine (mg/dL)	1.16 (0.47)	1.39 (0.97)	0.059
Preprocedural eGFR < 60 mL/min	46 (33)	57 (56)	0.212
Preprocedural dyslipidemia	94 (67)	85 (83)	0.081
Preprocedural AAA	8 (6)	11 (11)	0.614
Preprocedural carotid artery stenosis >50% or prior CEA	23 (16)	36 (35)	0.089
Preprocedural symptomatic PAD	18 (13)	23 (23)	0.452
Prior CABG	34 (24)	34 (33)	1.000
Prior PCI	32 (23)	42 (41)	0.261
Prior permanent pacemaker	8 (6)	14 (14)	0.336
Prior aortic valvuloplasty	17 (12)	24 (24)	0.259
Cardiovascular pharmacology			
ACE inhibitor	59 (42)	15 (15)	<0.001
Angiotensin II receptor blocker	41 (29)	2 (2)	<0.001
Beta blocker	83 (59)	81 (79)	0.841
Calcium channel blocker	24 (17)	24 (24)	1.000
Thiazide diuretic	25 (18)	15 (15)	0.118
Loop diuretic	55 (39)	51 (50)	0.642
Spironolactone	3 (2)	3 (3)	1.000
Statin	77 (55)	67 (66)	0.170
Aspirin	80 (57)	78 (76)	0.707
Dual antiplatelet therapy	20 (14)	33 (32)	0.080
Any anticoagulant	18 (13)	23 (23)	0.452

Values are mean (standard deviation) or % (n).

**Table 2:** Procedural Characteristics

	**RAAS (n=71)**	**Control (n=98)**	**P value**
Approach			
Transfermoral	76 (54)	72 (71)	.723
Transapical	18 (13)	26 (25)	.351
Transaortic	6 (4)	1 (1)	.163
Trans-subclavian	0 (0)	1 (1)	1.000
Mean LOS after TAVR (days)	5.3 (12.1)	4.2 (4.0)	.641
Valve type			
First generation Sapien	38 (27)	48 (47)	.213
Sapien XT	28 (20)	17 (17)	.131
Sapien S3	11 (8)	12 (12)	1.000
First generation CoreValve	20 (14)	19 (19)	1.000
CoreValve Evolute	3 (2)	3 (3)	1.000
Mean valve size (mm)	25.9 (2.9)	26.0 (2.8)	.947

Values are mean (standard deviation) or n (%).

**Table 3:** Echocardiographic data

	**RAAS **	**Control**	***P***
Preprocedural			
Aortic valve area (VTI) (cm^2^)	0.96 (0.34)	0.91 (0.25)	0.223
Peak aortic velocity (cm/s)	412.2 (63.4)	407.8 (61.8)	0.660
Peak aortic gradient (mmHg)	69.5 (20.8)	67.9 (19.3)	0.609
Mean aortic gradient (mmHg)	44.2 (13.3)	43.5 (12.1_	0.720
Ejection fraction (%)	55.6 (14.1)	58.6 (11.6)	0.131
Stroke volume (mL)	93.1 (25.9)	86.9 (17.5)	0.089
Moderate aortic regurgitation (%)	25 (18)	22 (21)	0.584
Severe aortic regurgitation (%)	4	4	1.000
Moderate mitral regurgitation (%)	37	18	0.012
Severe mitral regurgitation (%)	0	6	0.040
24 hour post-TAVR			
Aortic valve area (VTI) (cm^2^)	2.31 (0.75)	2.16 (0.65)	0.178
Peak aortic velocity (cm/s)	221.0 (53.1)	214.0 (54.6)	0.411
Peak aortic gradient (mmHg)	20.6 (10.1)	19.2 (10.9)	0.402
Mean aortic gradient (mmHg)	12.1 (5.9)	11.6 (7.0)	0.684
Ejection fraction (%)	61.0 (13.5)	61.3 (12.5)	0.886
Stroke volume (mL)	100.7 (28.6)	92.8 (28.9)	0.082
Moderate aortic regurgitation (%)	6	10	0.399
Moderate mitral regurgitation (%)	10	16	0.262
Severe mitral regurgitation (%)	0	4	0.140
1 year post-TAVR			
Aortic valve area (VTI) (cm^2^)	2.01 (0.62)	1.98 (0.60)	0.795
Peak aortic velocity (cm/s)	223.3 (49.6)	219.2 (50.8)	0.641
Peak aortic gradient (mmHg)	21.1 (9.0)	20.5 (11.1)	0.743
Mean aortic gradient (mmHg)	11.8 (5.0)	11.8 (6.6)	0.991
Ejection fraction (%)	58.0 (13.8)	58.1 (12.4)	0.948
Stroke volume (mL)	96.9 (29.4)	90.6 (28.1)	0.229
Moderate aortic regurgitation (%)	10	16	0.328
Moderate mitral regurgitation (%)	8	17	0.142
Severe mitral regurgitation (%)	2 (1)	9 (7)	0.138

Values are mean (standard deviation) or %.

**Table 4:** Primary and Secondary Outcomes

	**RAAS**	**Control**	***P***
% Survival > 1 year	100 (71/71)	100 (98/98)	1.000
% Survival > 2 year	95 (38/40)	79 (48/61)	0.042
Periprocedural major vascular	7 (5)	5 (5)	0.744
Periprocedural minor vascular	7 (5)	7 (7)	1.000
Post-TAVR PPM implantation	11 (8)	10 (10)	1.000
Periprocedural increase in serum creatinine >1.5x baseline	3 (2)	7 (7)	0.306
In Hospital			
MI	0 (0)	1 (1)	1.000
Stroke/TIA	1 (1)	0 (0)	0.420
HF exacerbation	21 (15)	20 (20)	1.000
All-cause mortality	0 (0)	0 (0)	1.000
Discharge to 30 days			
MACCE	18 (13)	21 (21)	0.699
Myocardial Infraction	3 (2)	1 (1)	0.573
Stroke/TIA	0 (0)	0 (0)	1.000
HF exacerbation	17 (12)	20 (20)	0.691
Rehospitalization for any reason	18 (13)	21 (21)	0.699
All-cause Mortality	0 (0)	0 (0)	1.000
30 days- 6 months			
MACCE	18 (13)	23 (23)	0.452
Myocardial Infraction	0 (0)	2 (2)	0.510
Stroke/TIA	3 (2)	1 (1)	0.573
HF exacerbation	8 (6)	21 (21)	0.032
Rehospitalization for any reason	17 (12)	23 (23)	0.340
All-cause mortality	0 (0)	0 (0)	1.000
6 months-1 year			
MACCE	21 (15)	33 (32)	0.118
Myocardial Infraction	3 (2)	2 (2)	1.000
Stroke/TIA	1 (1)	2 (2)	1.000
HF exacerbation	15 (11)	23 (23)	0.245
Rehospitalization for any reason	20 (14)	32 (31)	0.112
All-cause Mortality	0 (0)	0 (0)	1.000

Values are % (n). MACCE = major adverse cardiovascular and cerebrovascular events, defined as death from any cause, myocardial infarction, rehospitalization, and stroke.

## Discussion

This study from a predominantly rural area gives evidence to suggest that the use of that RAS blockade therapy with ACEIs or ARBs is associated with improved intermediate-term survival after TAVR for severe aortic stenosis. Furthermore, this study reaffirms the findings seen in similar studies, and endorses the external validity of the larger database studies. This trial also provide data to suggest that RAS blockade may also decrease the risk of heart failure exacerbation in the immediate post-operative period. Therapy with ACEIs or ARBs did not lead to any significant improvement in left ventricular function or stroke volume after TAVR, which is consistent with other prior studies.^14^ As expected, RAS blockade therapy does non-significantly decrease the prevalence of moderate and severe mitral regurgitation after TAVR, although the clinical significance of this is unknown.

The findings of this study are important and potentially intervenable given the changes in vascular hemodynamics after TAVR which have been noted in other studies. A 2015 study by Yotti et al found that systolic and pulse arterial pressures, vascular resistance, arterial elastance were significantly altered after TAVR.^15^ Furthermore, in a small study by Perlman et al, it was discovered that 51% of post-TAVR patients developed new onset or worsening hypertension within 5 days following TAVR.^16^ The results of these previous studies and the data from this trial underlines the importance of the monitoring and treatment of hypertension after TAVR.

Given the improvement in both short and long-term outcomes following TAVR, therapy with an ACEI or ARB may be indicated in all preexisting and new onset hypertension after TAVR. This is especially true given the widely known benefits of these agents in disease states that are commonly comorbid with significant aortic valve stenosis. With the data we report in this study, similar studies showing comparable results, and the 10-year data reported in the Goel et al study, we believe that it is highly likely that the benefit of ACEI/ARB demonstrated in these studies can be extrapolated in most clinical settings.^7^

Despite these encouraging findings, the mechanisms by which RAS blockade therapy improves survival remains unknown. Our study did not show any significant changes in echocardiographic parameters as originally hypothesized. Also, the non-significant trend towards reduced mitral regurgitation is not likely to be of any clinical significance since other studies have not showed mitral regurgitation to be a negative prognostic marker after TAVR.^17^ Given our findings, changes in endothelial function with ACEI or ARB is the single most likely mechanism by which RAS blockade therapy improves clinical outcomes.^18^ Additionally, optimizing the therapy of other patient comorbidities with ACEIs or ARBs likely also plays an important role in improving the overall outcome after TAVR.

The clinical setting in which this study was completed is significant in that there have been numerous studies outlining the deficiencies in cardiovascular care in rural areas.^19,20^ This disparity, compounded by the well-known changes in hemodynamics after TAVR, makes patient monitoring and follow-up both vital for successful post- TAVR outcomes and a major clinical challenge moving forward. Patients in rural areas often are older, have a higher burden of significant comorbidities, and receive less primary and secondary prevention, which limits the broad generalization and external validity of studies primarily completed in urban settings.^19^ As TAVR continues to evolve and medical centers offering TAVR extend beyond urban medical centers, continued evaluation of the safety of this procedure and the pharmacology incident to TAVR will be imperative.

Limitations of this study including its small sample size, single center experience, retrospective design, and inequalities in the length of post-procedural follow-up. The potential for other important confounding factors which were not part of this study’s baseline patient characteristics does exist, which is common among studies of this design. However, this study was intended to include all relevant baseline characteristics to successfully isolate the independent variable of interest. Patients in both groups were reasonably well matched overall, although there was an increase prevalence of diabetes mellitus in the cohort receiving ACEI or ARB. The presence of this baseline difference is very unlikely to impact the findings of our study, in that a history of diabetes mellitus was associated with worse clinical outcomes in other studies.^21^ There also was a significant increase in the prevalence of preprocedural hypertension in the RAS blockade cohort. Once again, we are not aware of any data which suggests that preprocedural hypertension is a prognostic marker for positive or negative outcomes after TAVR.^22^

## Conclusion

In this study from a predominately rural area, an association between the use of RAS blockade therapy with ACEIs and/or ARBs after TAVR and increased overall survival at 2 years was found. Additionally, the use of RAS blockade therapy post-TAVR was also associated with a trend towards decreased postprocedural heart failure exacerbations. These findings build upon previously published data and further suggests that TAVR may be a compelling indication for the use of an ACEI or ARB in all patients.

## Competing interests

BK has no relationships with industry to disclose.

## Ethical approval

This study protocol received dual IRB approval from the University of North Dakota IRB and from the Sanford Health IRB.

## Funding

This research did not receive any specific grant from funding agencies in the public, commercial, or not-for-profit sectors.

## Acknowledgments

The investigator would like to thank Thomas Haldis, DO and Cornelius Dyke, MD for their help in getting this project started, and Ronda Bolgrean, RN for her help with data acquisition.
